# Biofilm development during the start-up period of anaerobic biofilm reactors: the biofilm *Archaea* community is highly dependent on the support material

**DOI:** 10.1111/1751-7915.12115

**Published:** 2014-02-25

**Authors:** Frédéric Habouzit, Jérôme Hamelin, Gaëlle Santa-Catalina, Jean-P Steyer, Nicolas Bernet

**Affiliations:** Laboratoire de Biotechnologie de l'Environnement, Institut National de la Recherche AgronomiqueNarbonne, 11100, France

## Abstract

To evaluate the impact of the nature of the support material on its colonization by a methanogenic consortium, four substrata made of different materials: polyvinyl chloride, 2 polyethylene and polypropylene were tested during the start-up of lab-scale fixed-film reactors. The reactor performances were evaluated and compared together with the analysis of the biofilms. Biofilm growth was quantified and the structure of bacterial and archaeal communities were characterized by molecular fingerprinting profiles (capillary electrophoresis-single strand conformation polymorphism). The composition of the inoculum was shown to have a major impact on the bacterial composition of the biofilm, whatever the nature of the support material or the organic loading rate applied to the reactors during the start-up period. In contrast, the biofilm archaeal populations were independent of the inoculum used but highly dependent on the support material. Supports favouring Archaea colonization, the limiting factor in the overall process, should be preferred.

## Introduction

Anaerobic digestion is widely used for treating biodegradable agro-industrial waste and wastewater. This process, which allows the conversion of organic matter into methane (CH_4_) and carbon dioxide (CO_2_), is carried out by a complex anaerobic microbial consortium. Bacteria first convert complex organic matter into acetate, hydrogen (H_2_) and CO_2_, which are then converted into CH_4_ by methanogenic archaea. Methanogenesis involves slow-growing micro-organisms and it is generally considered as the limiting reaction in the anaerobic digestion process (Michaud *et al*., 2005). The failure of the start-up of full-scale anaerobic digesters is often linked to low activity of methanogenic archaea. These populations are very sensitive to any disturbance in anaerobic digesters, such as organic overload, leading to an accumulation of volatile fatty acids (VFA) and H_2_, which compounds are known to inhibit the process (Leitao *et al*., 2006).

The intensification of anaerobic digestion processes was achieved some decades ago by the development of biofilm-based or granule-based processes. Technologies allowing the decoupling of a short hydraulic retention time (HRT) and a long biomass retention time have been proposed for the treatment of organic effluents and biofilm reactors are able to maintain high concentrations of active microbial populations having low growth rates, such as *Archaea* (Escudié *et al*., 2005). The initial biofilm formation on the solid support is responsible for the long period needed for start-up (Lauwers *et al*., 1989; Perez *et al*., 1997). It is possible to reduce the time needed for biofilm formation using various strategies (Escudié *et al*., 2011). For example, a short HRT of less than 1 day induces a quick washout of the suspended biomass from the reactor, drastically improving biofilm growth on the carrier (Cresson *et al*., 2008).

Biofilm formation starts with adhesion of microbes to a surface, which relies on the physico-chemical characteristics of solids (support and micro-organisms) and liquids. In the literature, a large variety of materials has been used as support to immobilize micro-organisms. The support material determines biomass retention capacity (Alves *et al*., 1999), and the performance of the system is dependent on adherence phenomena. The physico-chemical characteristics of the support material are decisive parameters; the choice of the correct support is crucial for ensuring the success of the process (Garcia *et al*., 2008). The choice of the support material first impacts the initial adhesion of micro-organisms, quantitatively but also qualitatively; for example, polyvinyl chloride (PVC) was recently shown to promote initial adhesion of methanogenic *Archaea* (Habouzit *et al*., 2011). In this recent study, we showed that the use of low-surface free-energy materials facilitates the first stage of colonization (i.e. early adhesion) by anaerobic micro-organisms. According to the type of material, micro-organism adhesion is quantitatively different. There is also a strong difference in adhesion between *Bacteria* and *Archaea*. The nature of the support material can promote adhesion of specific groups of micro-organisms and can therefore be used as a design parameter to enhance biofilm formation in industrial reactors. In anaerobic digestion, the good performances generally observed in biofilm processes using PVC material as the biomass support could be explained by a high affinity for this material of methanogenic archaea, provided that this advantage in early adhesion is maintained during subsequent stages of biofilm growth.

The purpose of this new work was thus to study the potential impact of the nature of the support material not on the early microbial adhesion, but on the colonization of the support.

The originality of this study is in the link established between the overall performance during the start-up of lab-scale anaerobic reactors and measurement of the consortium described as two main microbial groups (*Archaea* and *Bacteria*) by using molecular tools. The molecular fingerprinting profiles highlight the relative abundance of the whole community. Throughout the experiments, VFA and soluble chemical oxygen demand (COD_s_) where used as indicators of process performance. Because of a fast washing-out of the suspended biomass obtained by a short HRT applied to the reactors, the methane yield (Y_CH4_) was considered as an indicator of biofilm formation, as proposed previously (Michaud *et al*., 2002). Molecular tools targeting *Archaea* and *Bacteria* were used for quantifying the adhesion and the growth of the biomass.

## Results and discussion

The reactors were operated for a period ranging from 26 to 55 days, depending on their performances according to the start-up strategy described below.

### Dynamics of organic loading rate (OLR) and COD removal efficiency

The reactors were started at a low initial OLR of about 0.5 g COD L^−1^ d^−1^ and at a HRT of 18 h. For each of the four experiments (*C1* to *C4*), maximum OLR varied according to the materials tested.

In each reactor, the same scenario was observed as follows: after inoculation, washout of suspended biomass occurred, during which outlet COD was higher than inlet COD and COD removal remained very low for several days. After this period of acclimatization of micro-organisms to their new environment, the COD removal rate increased to 80%, which meant that the OLR could be increased. If the COD removal rate stayed stable despite an increase in the OLR, the system would still be considered in its start-up phase because it had not yet reached its maximum loading rate. If the removal rate fell down below 80%, the increase in the OLR had to be stopped.

Reactors performances are compared in Table 1, based on the adaptation period and total start-up period. The performances obtained at the end of the start-up periods in terms of OLR and removal efficiencies are in the range of previous studies on similar processes (Ouichanpagdee *et al*., 2004 and Thanikal *et al*., 2007). The adaptation period is here defined as the time necessary to reach 80% COD removal at the initial OLR applied. The duration of start-up is the time needed to reach the maximum OLR while keeping COD removal efficiency at 80%.

**Table 1:** Quantitative parameters for the fixed-bed comparison

	C 1		C 2		C 3		C 4			
Period	PE	PP	Bf30	PVC	PP	PVC	PE	Bf30	Unit	
Adaptation	Duration	28	33	5	6	6	7	10	11	day
	COD_rem._ Rate^a^	1.5	2.25	1.1	0.97	2.1	2.4	0.63	0.67	gCOD_rem_ L^−1^ d^−1^
	(VSS)^a^	0.1	0.1	0.2	0.2	0.1	0.3	0.2	0.2	g L^−1^
	(VFA)^a^	0.2	0.3	0.15	0.25	0.2	0.75	0.1	0.1	g L^−1^
Start-up	Duration	35	55	11	34	31	31	19	13	day
	COD_rem._ Rate^a^	2.6	10	1.9	21.3	30	30	1.1	1	gCOD_rem_ L^−1^ d^−1^
	(VSS)^a^	0.05	0.3	0.1	0.1	0.4	0.8	0.1	0.15	g L^−1^
	(VFA)^a^	0.2	0.	0.3	0.2	0.2	0.2	0.3	0.1	g L^−1^
	Removal efficiency^a^	83	88	80	96	97	97	82	80	% COD

a Values measured at the end of each period.

In our experiments, final OLR values applied to the reactors were very different and up to 30 g L^−1^ d^−1^. In the first experiment (C1), the start-up period was long (55 days) because the polyethylene (PE) reactor could not reach an OLR higher than 3 g L^−1^ d^−1^ with 83% efficiency after 35 days of operation. In the second experiment (C2), the difference between the reactors was really significant: after 11 days, the reactor filled with Bioflow 30® (Rauschert Verfahrenstechnik GmbH, Steinwiesen, Germany), called Bf30 in this study, was not able to reach 80% COD removal despite a very low OLR. The OLR could be increased more rapidly when the reactors in this comparison were both filled with the best carriers of the C1 and C2 experiments (see Table 3). In the case of the polypropylene (PP) and PVC carriers, higher OLR was observed (30 g L^−1^ d^−1^).

### Evolution of suspended solids (SS)

The SS concentration in the effluent in the early phase of the start-up period gives an indication on the level of biomass washout from the reactors. Initially, the suspended biomass in the reactor is inoculum (around 1 gVSS L^−1^). Then, it was washed out after 4 to 5 HRT by the continuous liquid flow. In most cases, the decrease of volatile suspended solid (VSS) concentration continued through all the period studied (see [VSS] in Table 1). However, in C1 and C3 experiments, the VSS concentration increased in the last period (to 0.6; 0.4; 0.4 and 1 gVSS L^−1^ for PE-C1; PP-C1; PP-C3 and PVC-C3 respectively). This increase is due to either biomass detachment from the biofilm or growth of planktonic micro-organisms. The detachment is generally considered as proportional to the colonized surface and thus increases during the start-up period. Quantitative polymerase chain reaction (qPCR) analyses revealed that *Archaea* fraction is always higher in the biofilm compared with suspended biomass: the biofilm/liquid ratio of archaea fraction varies from 1.3 to 6.1. As slow-growing organisms, methanogens were probably present in the inner part of the biofilm and therefore protected from detachment. Moreover, an HRT of 18 h is compatible with the planktonic growth of bacteria. The VSS concentration increased at the end of experiments only when OLR was very high (e.g. experiment C3 with PP and PVC supports).

### Evolution of the VFA concentration

At the beginning of each experiment, VFA concentration in the reactors was very low (generally not detectable) because the inoculum sludge contained no VFA. During the adaptation period, the VFA concentration increased (Table 1), which was related to low removal efficiency due to the low growth rate of methanogenic archaea compared with acidogenic bacteria. Ethanol conversion to acetate was not followed by its transformation into methane. When the methanogenic activity became higher, the VFA concentration, mainly as acetate, started to decrease to its initial value. At higher OLR, higher VFA concentrations in the reactors made it possible to visualize the difference of activity between methanogenic *Archaea* and acidogenic populations. As soon as such transformation occurred, the removal efficiency increased with the VFA consumption. This corresponded to a good balance between the acidogenic and methanogenic populations. This balance was disturbed at each increase of the OLR. If the increase was small (10–20%), a new equilibrium was attained after a single residence time. The OLR reached its maximum value when the biofilm could not grow further. The start-up was then completed. When equilibrium was not restored, the VFA concentration increased. The methanogenic community was not able to consume the VFA produced, which thus accumulated. This was often the case at the end of our experiments when the VFA represented almost the totality of effluent COD.

### Evolution of biogas production and methane yield

Biogas flow rate and composition are key parameters in the monitoring of an anaerobic reactor. From biogas production and COD removal (COD_rem_), it is possible to calculate the methane yield, which expressed in litre of biogas per gram of COD_rem_, enables the dynamics of biofilm formation during start-up to be followed.

During the establishment of the biofilm the, methane yield (Y_CH4_) can be related to microbial activity (i.e. quantity of COD removed) as the result of the balance between the flows of organic carbon to catabolism and anabolism in methanogenic ecosystems (Michaud *et al*., 2005). In stable conditions, its value is constant; in theory 0.35 L_CH4_ gCOD_rem_^−1^ (STP). Therefore, as shown previously by (Michaud *et al*., 2002), the methane yield starts to increase during the lag phase to reach a stable value at the end of the start-up period.

**Figure 1:**
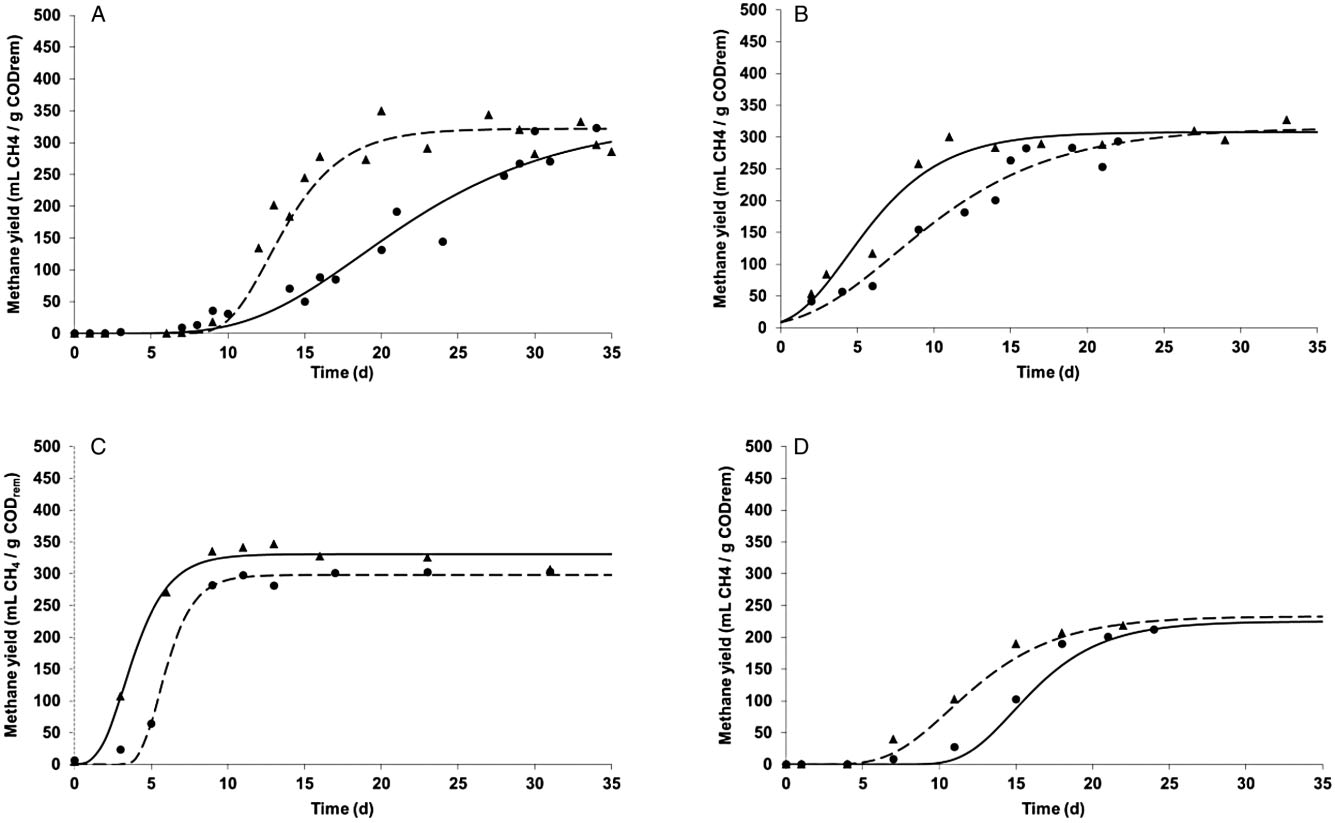
Evolution over time of methane yield during experiments C1 (A: •, PE; ▴, PP; 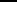, Gompertz PE; 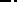, Gompertz PP), C2 (B: ▴, PVC; •, Bf30; 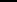, Gompertz PVC; 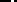, Gompertz Bf30), C3 (C: ▴, PVC bis; •, PP bis; 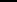, Gompertz PVC bis; 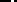, Gompertz PP bis) and C4 (D: ▴, PE bis; •, Bf30 bis; 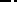, Gompertz PE bis; 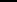, Gompertz Bf30 bis).

Figure 1 shows the dynamics of the methane yield in the four comparative studies. The timeline of Y_CH4_ accounts for the three phases of biofilm formation: induction, growth and steady state. The use of the modified Gompertz equation allows modelling the growth of a regulated population as previously described (Quéméneur *et al*., 2012). Details of this equation are provided as supplementary online information (see Supporting Information Text S1). In all cases, Y_CH4_ is very low at the beginning of the start-up period, indicating strong anabolic activity of the micro-organisms. This value then increases up to a stable level corresponding to the end of biofilm formation. Y_CH4_ followed the same sigmoidal profile during the evolution of the eight reactors, similar to a classic exponential bacterial growth curve.

The proportion of methane in the biogas increased then stabilized, reaching values from 70% to 90%. The evolution of the production of biogas varied according to the organic load and COD removal efficiency. It was very low during the early days but increased in proportion to the increase in the flow and the OLR. In Table 2, the parameters obtained from the Gompertz equation with the different supports are compared.

**Table 2:** Gompertz parameters calculated to model Y_CH4_ progress in the four experiments

	C1		C2		C3		C4	
PE	PP	Bf30	PVC	PP	PVC	PE	Bf30	
Maximum Y_CH4_	0.335	0.322	0.315	0.308	0.298	0.331	0.233	0.225
(L_CH4_ g COD_rem_^−1^)	±0.010	±0.006	±0.034	±0.012	±0.006	±0.007	±0.011	±0.012
Time for maximum Y_CH4_	30	23	15	10	9	9	∞	∞
(Days)								
MIR	0.017	0.045	0.02	0.033	0.089	0.076	0.0322	0.026
(L_CH4_ g COD_rem_^−1^)	±0.002	±0.009	±0.003	±0.006	±0.022	±0.013	±0.002	±0.004
λ^a^	11.4352	10.004	1.637	0.926	4.287	1.612	6.761	11.615
(days)	±1.1043	±0.846	±1.208	±0.849	±0.246	±0.362	±0.515	±0.5920
R^2^	0.965	0.956	0.951	0.948	0.994	0.989	0.995	0.992
(%)								

MIR, maximum increase rate of the methane yield.

a Lag-phase time.

**Table 3:** Characterization of attached and suspended biomass at the end of the experiments

	C1		C2	C3		C4		Unit	
	PE	PP	Bf30	PVC	PP	PVC	PE	Bf30	
Attached biomass	18.39	78.37	9.97	98.38	169.9	176.2	4.31	6.71	gVSS
Suspended biomass	12.95	6.35	34.2	26.80	61.38	52.39	11.83	5.36	gVSS
COD_rem._ rate	2.60	10.00	1.90	21.26	30.00	30.00	1.14	1.02	gCOD_rem_ L^−1^ d^−1^
Biofilm production	11.06	41.41	27.50	31.44	37.85	41.91	8.45	19.72	gbiofilm kgCOD_rem_^−1^

Values of methane yield remain close to those of the literature (Michaud *et al*., 2002; Michaud *et al*., 2005; Cresson *et al*., 2006). They do not vary much (4.4 %). The mean value was around 318 L gCOD^−1^ except for experiment C4 in which methane yield values were lower than expected (0.229 ± 0.004 L gCOD^−1^).

According to the values of methane yield obtained, it appears that some carriers do not facilitate the adhesion of a stable anaerobic ecosystem. Some values obtained were close to the theoretical value which is 0.35 LCH4 gCODrem−1 in standard conditions for temperature and pressure (STP) (Hickey and Owens, 1981; Balaguer *et al*., 1997) but for others (PE, Bioflow 30®) the theoretical value was not always reached and when this was the case, the time necessary to reach this maximum was long.

The kinetics of methane yield can be used to compare the different start-up experiments. During the first phase, Y_CH4_ was very low because methanogens where not active and a major part of the metabolism of the micro-organisms was directed to the production of biomass. Eventually, the methane yield increased. Several authors report the observation of such a lag phase in the production of methane (Lauwers *et al*., 1990 and Sanchez *et al*., 1994). The time required to reach the maximum methane yield illustrates the effect of the particular support material. Furthermore, in one experiment, when both materials facilitated the adhesion of the ecosystem, the rapid increase of the OLR was possible and reactor performances were very good (see C3 in Table 2). In the same way, the different results obtained for the same material in two different experiments could be explained by the profile of OLR increase, which was dependent on the reactor with the slowest evolution in removal efficiency.

### Biofilm quantification

At the end of each start-up experiment, 30 support modules were sampled randomly from the reactors. The dry matter was measured on each of them. The average of these 30 values was calculated and the total quantity of biofilm in the reactor was estimated according to the number of modules. The SS concentration was also measured in the liquid. The results are presented in Table 3 together with the COD_rem_ rate and biofilm production of biofilms in each reactor.

The highest biofilm concentrations were obtained in the reactors containing PP and PVC carriers. The biofilm mass, which adhered to the PP carriers at the end of the first experiment (C1), was three times as high as that fixed on PE carriers. In the second experiment, it was 10 times as high on the PVC as on the Bioflow 30®. In the third and fourth, the quantity of biofilm was equivalent in both reactors. In these experiments, neither the quantity of fixed biomass nor its activity permitted differentiating the carriers. In the first comparison, the big quantity of suspended biomass in the ‘PE’ reactor is explained by the high OLR imposed by the ‘PP’ reactor, which achieved better overall activity. This was also the case for reactor Bf30 in the second experiment.

The correlation between the quantity of total biomass and the COD removal rate of the reactor was strong (R^2^ = 0.97). This activity depended on the amount of attached biomass on carriers (R^2^ = 0.97) rather than on suspended biomass concentrations (R^2^ = 0.66). This observation can be explained by a higher *Archaea* concentration in the biofilm compared with the bulk with a percentage ratio biofilm/bulk ranging from 1.3 to 6.1 in the different experiments.

### Microbial community structure

**Figure 2:**
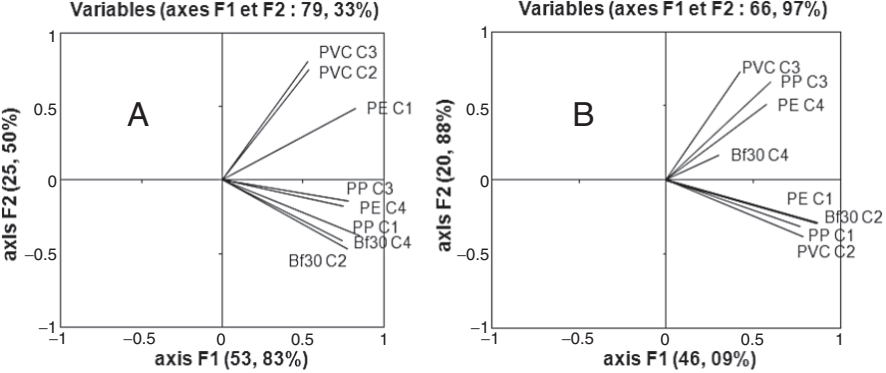
Principal component analysis ordination plot from archaeal (A) and bacterial (B) CE-SSCP profiles of adhered communities. The physical distances between points are proportional to the genetic distances of community profiles.

In parallel to the overall adhesion measurements as previously presented, the relative proportions of the dominant populations in the inoculum and in the attached biomass were determined for each support using capillary electrophoresis-single strand conformation polymorphism (CE-SSCP) fingerprinting, for both archaeal and bacterial populations. A mathematical procedure based on the Euclidian distances between profiles was carried out in order to highlight the genetic distance between the communities (Zumstein *et al*., 2000). Principal component analysis (PCA) is useful for discerning patterns within CE-SSCP data (Sen and Hamelin, 2008 and Quéméneur *et al*., 2012). The results of this PCA are presented in Fig. 2A for *Archaea* and Fig. 2B for *Bacteria*.

No direct link could be shown between the nature of the various materials and the distribution of the attached bacteria (Fig. 2B). For a given material and from one comparative experiment to another, the populations were different. The composition of the bacterial biofilm seems to have been driven mainly by the composition of the inoculum, whatever the nature of the support material or the OLR. The role of the inoculum during the adhesion of micro-organisms has been underlined in the literature (Annachhatre and Bhamidimarri, 1992; Michaud *et al*., 2002; Cresson *et al*., 2009). Considering the PCA for *Archaea* profiles (Fig. 2A), the results are totally different. The biofilm archaeal populations were independent of the type of inoculum used but highly dependent on the support material, except for the PE support on which biofilm formation was not successful and whose results are therefore not significant.

The study of the colonization of four support materials made it possible to highlight differences related to the type of materials. Indeed, depending on the support material, the time required to obtain a well-established biofilm can vary greatly. PP and PVC have been shown to be the best materials for obtaining efficient biofilm formation in a relatively short period of time. This result should be analyzed in relation to our previous study in which these two materials were shown to favour initial *Archaea* adhesion (Habouzit *et al*., 2011). This early advantage seems to persist during the start-up period. Thus, a 2 h test could be applied to select an efficient support material.

It must be emphasized that the crucial role played by the material can be masked by the mode of operation of a reactor, especially during the start-up period. This fact explains why, for the same material, the results obtained can be very different in terms of performance and colonization.

## Experimental procedures

### The materials used as carrier

Four different carrier materials were used, based on the geometry of the Bioflow 30® model (Supporting Information Fig. S1 is available online), a commercial PE carrier used in moving bed biofilm reactors. The three new carriers have been produced by the Wintex Company (Coimbatore, India). These carriers are made of PE, PP and PVC. These varieties of materials that have been currently used as a support to immobilize anaerobic micro-organisms were chosen to determine biomass retention capacity.

### Design of the lab-scale anaerobic fixed-bed reactors

Each of the experiments was carried out successively in two replicated anaerobic fixed-bed reactors, height 56 cm and diameter 20 cm. The reactors (Supporting Information Fig. S2 is available online) were made of PVC with an active volume of 15.1 L and were maintained in mesophilic conditions at 37°C in a water bath. The reactors were operated in a continuous mode (20 L d^−1^) and the feed was pumped into the bottom of each reactor by means of a Masterflex® peristaltic pump (Cole-Parmer, Vernon Hills, IL, USA). The liquid inside the reactors was homogenized with immersed pump at the rate of 66 L min^−1^. At the top of each reactor, an outlet port was made connected through a U-tube for separation of biogas. pH and temperature were measured online at the top of each reactor.

The reactors were filled with 230 carriers, representing a total specific surface of about 2.56 m^2^. The biogas production rate was measured online by an Aalborg mass flow meter (0–50 ml min^−1^) fitted with a 4–20 mA output or by Milligascounter MGC-1 flow meters (Ritter, Bochum, Germany) at 25°C and local atmospheric pressure, depending on the gas flow. The ‘modular spc’ software, a home-made data acquisition system, was used for data acquisition (biogas production rate and pH).

### Feeding characteristics and reactor inocula

Sludge from a lab-scale anaerobic reactor originally fed with diluted wine was used as the source inoculum for the reactors. The sludge was diluted to an initial VSS concentration of 1 ± 0.05 g l^−1^. The specific COD removal rate of this inoculum, measured using ethanol as a substrate, was 0.6 ± 0.2 gCOD_rem_ gVSS^−1^ d^−1^.

Diluted red wine was used as the carbon source. It was supplemented with nutrients (ammonium hydrogenophosphate and ammonium chloride), corresponding to a COD/N/P ratio of 400/7/1. Deficiency in trace elements was prevented by the addition of a micronutrient solution (Cresson *et al*., 2006).

### Physico-chemical analyses

Total suspended solids (TSS), VSS and COD were analysed in accordance with the Standard Methods for examination of water and wastewater (APHA, 1992). The biomass of the biofilm within the reactor was determined at the end of the experiment by measuring the amount of solids attached to the carriers by drying samples for 24 h at 105°C.

VFA concentrations and soluble COD of the discharged effluents were determined through off-line analysis after 10 min centrifugation at 12 000*g* (corresponding to 9000 rpm with the rotor used). VFA were measured with a Varion 3900 flame ionization detector gas chromatograph (Agilent Technologies, Palo Alto, CA, USA). The column was a semi-capillary Econocap FFAP (Alltech Associates Inc., Deerfield, IL, USA). The temperature of the oven was programmed to rise from 80°C to 120°C with increments of 10°C min^−1^. The biogas composition was analyzed by gas chromatography (Shimadzu GC 8A, Nakagyo-ku, Kyoto, Japan) equipped with a CTRI Alltech column (Alltech Associates Inc., Deerfield, IL, USA). Detection was performed with a thermal conductivity detector. The measurement of CO_2_ and CH_4_ was obtained by internal calibration. Argon was used as the carrier gas.

### Start-up strategy (HRT and OLR)

To obtain rapid adhesion of the seeded micro-organisms to the carrier, the dilution rate must be higher than the maximum growth rate of the micro-organisms (Escudié *et al*., 2011). Thus, the short HRT applied to the reactors was set at a constant 18 h to facilitate the fast washing-out of the suspended biomass. The targeted daily increase of the OLR was 15%. However, this had to be adapted to the biofilm development, in conditions of a minimum COD removal efficiency of 80%, as described previously (Cresson *et al*., 2008). Each experiment consisted in a comparison of two similar reactors filled with a different carrier. An important point is that both reactors in an experiment were submitted to the same OLR profile, the increase of the OLR being driven by the reactor with the lowest performance.

### Microbial community structure

The microbial community structure of the *Bacteria* and *Archaea* organized in biofilms were analyzed after DNA extraction and polymerase chain reaction (PCR) amplification by CE-SSCP.

Microbial cells were harvested from the carriers with a 2% RBS35 solution (Société des Traitements Chimiques de Surface, Lambersart, France) at 50°C for 20 min with agitation and washed five times with a 9% NaCl solution at 50°C for 5 min. After centrifugation at 3000*g* for 5 min, pellets were stored at −80°C. Total genomic DNA was extracted and purified using a QiaAmp DNA stool mini kit, in accordance with the manufacturer's instructions (Qiagen, Hilden, Germany). Extractions were confirmed using Infinite 200 PRO NanoQuant (Tecan Group Ltd., Männedorf, Zwitzerland). The V3 regions of the 16S rRNA genes were amplified using the primers listed in Table 4 according to Braun and colleagues (2011). Amplification product sizes were quantified using the Bioanalyser 2100 (Agilent Technologies Gmbh, Böblingen, Germany).

**Table 4:** Sequences and target positions of the primers used in this study

Primer	Direction	Sequence (5′–3′)	Targeted 16S rRNA
W274	Forward Reverse	CCC TAC GGG GCG CAG CAG	*Archaea*
W275	Reverse	6-FAM-TTA CCG CGG CGG CTG	*Archaea*
W104	Reverse	TTA CCG CGG CTG CTG GCA C	Universal
W49	Forward	6-FAM-AGG TCC AGA CTC CTA CGG G	*Bacteria*

CE-SSCP analysis permits the separation of DNA fragments of the same size but with a different composition (Sen and Hamelin, 2008). Briefly, 1 μL of diluted PCR products was added to 18.925 μL of formamide and 0.075 μL of internal size standard Rox 400 HD (Applied Biosystems, Foster City, CA, USA). Each sample was then denatured for 5 min at 95°C and placed directly on ice for 10 min. CE-SSCP was carried out using the ABI 3130 genetic analyzer (Applied Biosystems) equipped with four 50 cm capillary tubes filled with 5.6% of conformation analysis polymer (Applied Biosystems) in the corresponding buffer along with 10% glycerol. The injection of DNA into the capillaries required 5 kV for 3 s. Electrophoresis was carried out at 15 kV and 32°C for 30 min per sample.

### Statistical analyses

PCA from the *archaeal* and *bacterial* CE-SSCP profiles of attached biomass was done using Xlstat software (Xlstat v7.5.2 Addinsoft, Paris, France). Bacterial fingerprints from the single strand conformation polymorphism profiles were aligned based on the ROX internal size standards and normalized using the StatFingerprints package (Michelland *et al*., 2010) in the collaborative software environment R (R Development Core Team since 2009).

## Conclusions

The nature of the support material affects the start-up of methanogenic biofilm reactors. PP and PVC supports showed the best results in term of maximum OLR and COD removal efficiency. The nature of the support material strongly impacts the structure of the microbial community of the biofilm. The bacterial species of the biofilm vary from one support to another and are highly dependent on the composition of the inoculum. In contrast, *Archaea* populations were shown to be specific to each material regardless of the inoculum.

## Supporting information

Text S1 The methane yield (YCH4) data for each reactor were fitted to a modified Gompertz Equation used by Zwitering *et al*.

Where Y_CH4_ is the methane yield (L_CH4_.gCOD_rem_ −1), MIR is the maximum increase rate of the methane yield (L_CH4_.gCOD_rem_^−1^.d^−1^), λ is the lag-phase time (days), t is the reaction time (days) and e is exp (1). The values of Y_CH4_, MIR and λ were estimated using a non-linear regression algorithm developed in Matlab (version 6.5, Mathworks). (Zwietering, M.H., Jongenburger, I., Rombouts, F.M. and van't Riet, K. (1990) Modeling of the bactérial growth curve. *Applied and environmental microbiology* **56**(6), 1875–1881.)

Fig S1 Original Bioflow 30® made of PE.

Fig S2 Lab-scale anaerobic fixed-bed reactor.
