# Supplementary material for: Biofilm development during the start-up period of anaerobic biofilm reactors: the biofilm Archaea community is highly dependent on the support material
Source: Microb Biotechnol. 2014 Feb 25;7(3):257–64. doi: 10.1111/1751-7915.12115 (PMC3992021; doi:10.1111/1751-7915.12115)
Supplement: Text S1 — The methane yield (YCH4) data for each reactor were fitted to a modified Gompertz Equation used by Zwitering et al. Where YCH4 is the methane yield (LCH4.gCODrem −1), MIR is the maximum increase rate of the methane yield (LCH4.gCODrem −1.d−1), λ is the lag-phase time (days), t is the reaction time (days) and e is exp (1). The values of YCH4, MIR and λ were estimated using a non-linear regression algorithm developed in Matlab (version 6.5, Mathworks). (Zwietering, M.H., Jongenburger, I., Rombouts, F.M. and van't Riet, K. (1990) Modeling of the bactérial growth curve. Applied and environmental microbiology 56(6), 1875–1881.) [file mbt20007-0257-sd1.docx]

The methane yield (Y_CH4_) data for each reactor were fitted to a modified Gompertz Equation used by Zwitering *et al.*

$$Y_{CH4}(t)=Y_{CH4}. exp\left[ -exp(\frac{MIR . e}{Y_{CH4}}\left( \lambda-t \right)+1 \right]$$

Where Y_CH4_ is the methane yield (L_CH4_.gCOD_rem_^-1^), MIR is the maximum increase rate of the methane yield (L_CH4_.gCOD_rem_^-1^.d^-1^), λ is the lag-phase time (days), t is the reaction time (days) and e is exp (1). The values of Y_CH4_, MIR and λ were estimated using a non-linear regression algorithm developed in Matlab (version 6.5, Mathworks).

Zwietering, M.H., Jongenburger, I., Rombouts, F.M. and van't Riet, K. (1990) Modeling of the bactérial growth curve. *Applied and environmental microbiology* **56**(6), 1875-1881.
