# Supplementary figures and images for: Biofilm development during the start-up period of anaerobic biofilm reactors: the biofilm Archaea community is highly dependent on the support material
Source: Microb Biotechnol. 2014 Feb 25;7(3):257–64. doi: 10.1111/1751-7915.12115 (PMC3992021; doi:10.1111/1751-7915.12115)

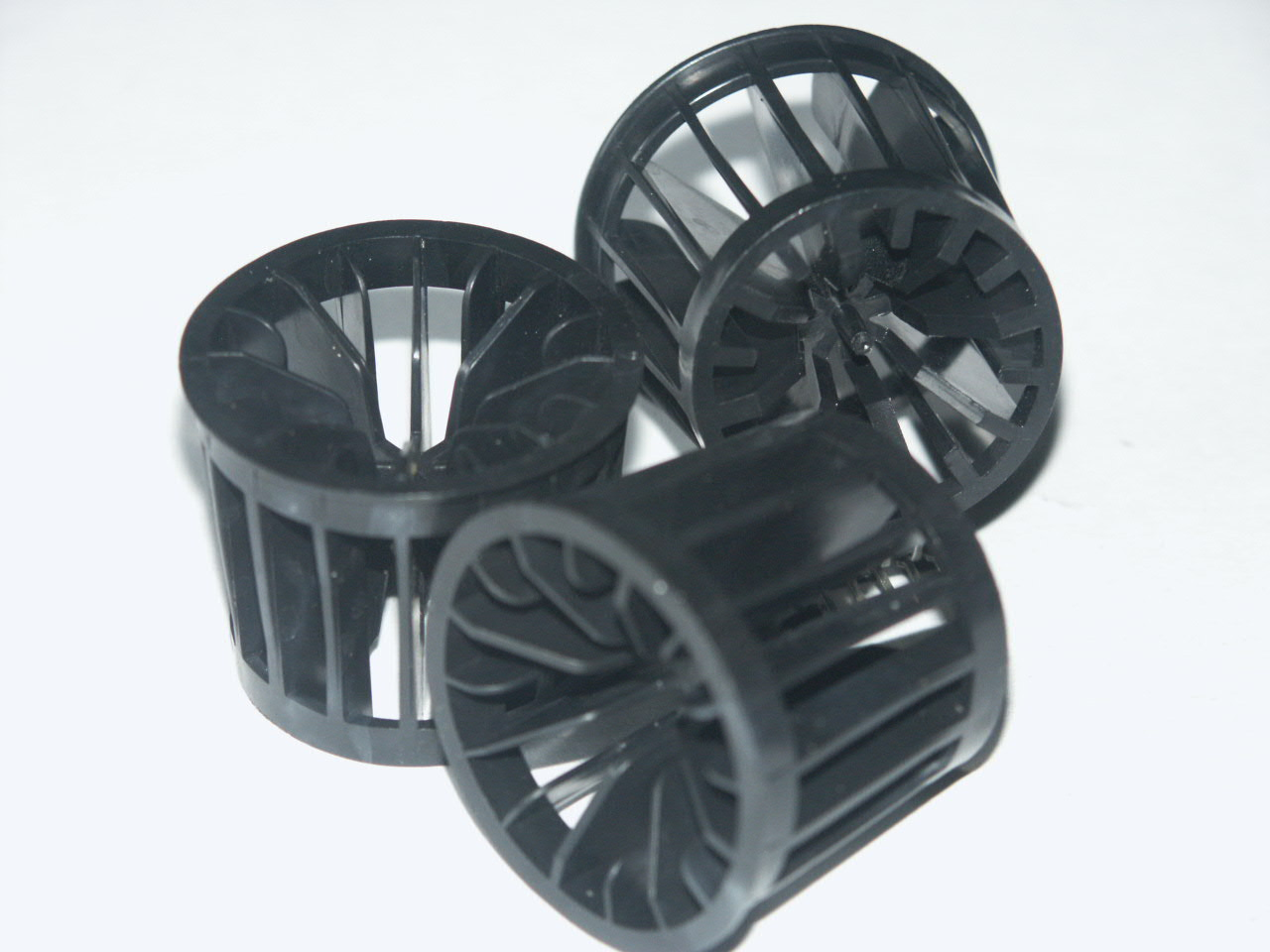

Supplement: Fig S1 — Original Bioflow 30® made of PE. [file mbt20007-0257-sd2.tif]

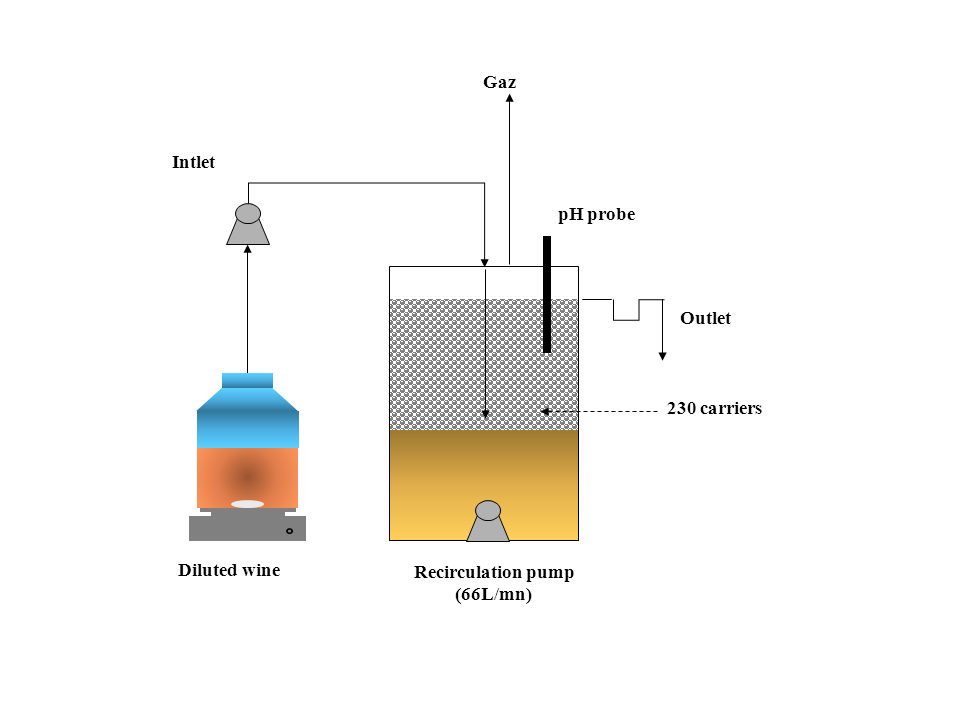

Supplement: Fig S2 — Lab-scale anaerobic fixed-bed reactor. [file mbt20007-0257-sd3.tif]
